# Inhibition of CBP/β-catenin and porcupine attenuates Wnt signaling and induces apoptosis in head and neck carcinoma cells

**DOI:** 10.1007/s13402-019-00440-4

**Published:** 2019-05-14

**Authors:** Robert Kleszcz, Anna Szymańska, Violetta Krajka-Kuźniak, Wanda Baer-Dubowska, Jarosław Paluszczak

**Affiliations:** Department of Pharmaceutical Biochemistry, Poznan University of Medical Sciences, ul. Święcickiego 4, 60-781 Poznań, Poland

**Keywords:** Head and neck cancer, Wnt pathway, Porcupine, CBP, IWP-2, PRI-724

## Abstract

**Purpose:**

Activation of the Wnt pathway contributes to the development of head and neck squamous cell carcinomas (HNSCC) and its inhibition has recently emerged as a promising therapeutic strategy. Here, we aimed at identifying suitable molecular targets for down-regulation of canonical Wnt signaling in HNSCC cells.

**Methods:**

Candidate target genes (*PORCN, WNT3A, FZD2, FZD5, LRP5, DVL1, CIP2A, SET, KDM1A, KDM4C, KDM6A, CBP, CARM1, KMT2A, TCF7, LEF1, PYGO1, XIAP*) were silenced using siRNA and selected targets were subsequently blocked using small molecule inhibitors. The effect of this treatment on the expression of β-catenin-dependent genes was assessed by qRT-PCR. The effect of the inhibitors on cell viability was evaluated using a resazurin assay in HNSCC-derived cell lines. A luciferase reporter assay was used for confirmation of the inhibition of Wnt-dependent gene expression. Cell migration was evaluated using a scratch wound healing assay. Cytometric analysis of propidium iodide stained cells was used for cell cycle distribution evaluation, whereas cytometric analysis of caspase 3/7 activity was used for apoptosis induction evaluation.

**Results:**

We found that inhibition of Porcupine and CBP/β-catenin interaction by IWP-2 and PRI-724, respectively, most strongly affected β-catenin-dependent gene expression in HNSCC cells. These inhibitors also induced apoptosis and affected HNSCC cell migration.

**Conclusions:**

Targeting Porcupine or the CBP/β-catenin interaction seems to be an effective strategy for the inhibition of canonical Wnt signaling in HNSCC cells. Further studies are required to confirm the possible therapeutic effect of IWP-2 and PRI-724 in HNSCC.

## Introduction

Head and neck squamous cell carcinoma (HNSCC) is the sixth most common cancer worldwide. Unfortunately, recurrence following conventional treatment, including surgery, radiotherapy, chemotherapy, or combined therapy occurs relatively frequently and recurrent tumors are associated with an unfavorable prognosis. The improvement of patient survival will require a deeper understanding of the molecular mechanisms underlying the pathogenesis of HNSCC [1–3].

The canonical Wnt signaling pathway has recently been identified as one of the cellular pathways that shows activation in HNSCC. Activation of this pathway may result from mutations in pathway-related genes, such as *FAT1* [4–8], as well as from aberrant methylation of genes that encode proteins acting as extracellular or intracellular antagonists of Wnt signaling, such as *SFRP1–5*, *WIF1*, *DKK1–3*, *DACH1* or *PPP2R2B* [7, 9–11]. It has also been reported that the expression of Wnt ligands, Frizzled receptors and Dishevelled, as well as β-catenin, is increased in HNSCC cells [7, 12–15]. Other evidence for activation of the Wnt pathway in HNSCC comes from the frequent detection of nuclear β-catenin and the enhancement of expression of β-catenin target genes, such as *c-MYC*, *CCND1*, *MMP7* or *survivin* (*BIRC5*) in HNSCC cells. Moreover, it has been reported that enhanced expression of β-catenin correlates with a shorter survival of patients with oral carcinomas [15]. In line with these observations, it has been found that knockdown of β-catenin reduces the growth of HNSCC cells and tumors [16, 17]. Animal models have shown that experimental induction of oral tumors by 7,12-dimethylbenz[a]anthracene or 4-nitroquinoline 1-oxide is associated with enhancement of Wnt signaling [18, 19]. These observations have led to the suggestion that Wnt inhibition may serve as a potential preventive and/or therapeutic strategy for oral carcinomas. Importantly, it has been found that cigarette smoke may induce Wnt signaling [15]. Activation of β-catenin signaling may also lead to an increased capacity of cells to invade or migrate [20]. Thus, β-catenin signaling may contribute to epithelial to mesenchymal transition (EMT) in HNSCC cells and, as such, may be associated with local recurrence and lymph node metastasis. Moreover, β-catenin may inhibit the differentiation of keratinocytes. Indeed, expression of β-catenin has been found to be higher in poorly differentiated tumors [15]. Importantly, nuclear β-catenin is, in contrast to oral leukoplakia, usually not detected in normal oral mucosa. Dysplastic leukoplakia usually shows a stronger nuclear accumulation of β-catenin than non-dysplastic leukoplakia [21] and it has been suggested that enhanced cell proliferation in oral epithelial dysplasia may be caused by enhanced Wnt signaling [22]. Thus, accumulating evidence indicates that activation of the Wnt signaling pathway is associated with the development and progression of head and neck cancers. This notion indicates that inhibition of this pathway may have therapeutic potential, not only for blocking HNSCC cell proliferation and invasion, but also for blocking the renewal of its cancer stem cells [23], which are believed to be responsible for recurrence. In particular, inhibition of this pathway may show efficacy towards higher grade, invasive tumors, which are currently difficult to cure.

As yet, few studies have explored the therapeutic potential of Wnt pathway inhibition in HNSCC and only few strategies for blocking Wnt signaling in HNSCC cells have been tested. Attenuation of Wnt ligand palmitoylation by blocking Porcupine using LGK974 has led to Wnt signaling inhibition in one third of HNSCC cell lines [24] and immunotherapy targeting Wnt ligands has been found to inhibit the proliferation of selected HNSCC cell lines [14]. Also, the potential of all-trans retinoic acid to induce differentiation and inhibit HNSCC cell growth may at least partly be ascribed to inhibition of Wnt signaling [25].

Here, we aimed at searching for molecular targets suitable for Wnt signaling inhibition among a wide set of genes associated with the multi-level regulation of Wnt signaling. We selected eighteen potential candidates (*PORCN, WNT3A, FZD2, FZD5, LRP5, DVL1, CIP2A, SET, KDM1A, KDM4C, KDM6A, CBP, CARM1, KMT2A, TCF7, LEF1, PYGO1, XIAP*) whose activity is crucially important for canonical Wnt signaling. The identification of targets whose down-regulation effectively inhibits Wnt signaling in HNSCC cells was followed by assessment whether their modulation significantly affects the proliferation, migration and apoptosis of HNSCC cells.

## Materials and methods

### Cell lines and culture

HNSCC cell lines derived from different anatomical locations (tongue: CAL27, SCC-25; hypopharynx: BICR6, FaDu; floor of mouth: H314, UM-SCC-1) were used. An immortalized normal keratinocyte (from floor of mouth)-derived cell line, OKF4/TERT-1, was used for comparison. The cell lines were purchased from the ATCC (CAL27, FaDu, SCC-25), the ECACC (BICR6, H314) or Merck (UM-SCC-1). The OKF4/TERT-1 cell line was obtained from prof. James Rheinwald’s Lab, Harvard Skin Disease Research Center, Harvard Medical School, USA.

CAL27, FaDu, BICR6 and H314 cells were grown in high-glucose DMEM medium (Biowest, France) supplemented with 10% FBS (EURx, Poland) and 1% antibiotics solution (Biowest, France). OKF4/TERT-1 cells were grown in Keratinocyte-SFM medium (Gibco, UK) supplemented with bovine pituitary extract, EGF (0.2 ng/ml), 0.4 mM CaCl_2_ and antibiotics solution. SCC-25 and UM-SCC-1 cells were grown in a 1:1 mixture of the aforementioned complete DMEM and complete Keratinocyte-SFM media. Standard incubation conditions (37 °C, 5% CO_2_, 95% humidity) were used for all cell lines.

### siRNA transfection

CAL27 and FaDu cells (1 × 10^5^/well) were seeded in 24-well plates and immediately transfected with 40 nM siRNA using jetPRIME transfection agent (Polyplus, France), according to manufacturer’s recommendations. For each gene, two distinct siRNA duplexes targeting different regions within the transcript sequence were used (Qiagen, Germany). Non-targeting siRNA was used as control. RNA was isolated 48 h post-transfection. The experiments were repeated twice.

### RNA isolation and quantitative real-time PCR (qRT-PCR)

Total RNA was isolated from cultured cells using a Universal RNA Purification Kit (EURx, Poland) according to the manufacturer’s recommendations. The quality and quantity of the RNA were assessed by spectrophotometry using a NanoDrop apparatus (Thermo Fisher Scientific, USA). In order to quantitatively analyze gene expression at the transcript level, cDNA was synthesized using a RevertAid First Strand cDNA Synthesis Kit (Thermo Fisher Scientific, USA), according to the manufacturer’s protocol. Hot FIREPol EvaGreen qPCR Mix Plus (Solis Biodyne, Estonia) was used to perform real-time PCR reactions using a LightCycler 96 apparatus (Roche, Germany) as reported before [26]. Each sample was run in triplicate and samples from two independent experiments were analyzed in parallel. The primer sequences for the expression analysis of β-catenin-target genes (*Axin2*, *BIRC5*, *CCND1*, *c-MYC* and *MMP7*) have been reported before [26]. The primer sequence used for analysis of the knock-down efficiencies of genes targeted by siRNAs are available upon request. Relative changes in transcript levels were calculated using the ΔΔCt method. The mean expression level of two reference genes (*TBP* and *PBGD*) was used for normalization.

### Inhibitors and resazurin viability assay

Eight inhibitors, GSK-J4, GSK-LSD1, IWP-2, ML324, MS049 (Sigma, USA), Dvl-PDZ Domain Inhibitor II (Calbiochem/Merck, USA), PRI-724 and MM-102 (Selleck Chemicals, USA), which selectively block the activity of KDM6A/B, KDM1A (LSD1), Porcupine, KDM4C, CARM1, Dishevelled, CBP/β-catenin and KMT2A, respectively, were used. Stock solutions of the compounds (20 mM; 8.75 mM in the case of IWP-2) were prepared in DMSO and stored in aliquots at −20 °C. A resazurin assay was performed to assess the effect of these compounds on the viability of HNSCC cells. Resazurin is converted by metabolically active cells into fluorescent resorufin and, thus, the level of the fluorescence signal is proportional to the number of viable cells. Briefly, cells were seeded in 96-well plates (10^4^ cells per well) and left overnight for adhesion. Next, the growth medium was replaced with fresh medium containing various concentrations of the test compounds and the cells were incubated for an additional 24 h. Subsequently, the cells were rinsed with PBS buffer after which fresh medium containing 1 μg/ml resazurin was added. After 2 h of incubation the level of fluorescence was measured (ex/em – 530/590 nm). The assay was performed at least twice, with four replicates per assay.

### Protein extraction and Western blotting

Cells (2.5 × 10^5^/well) were seeded in 6-well plates and, after overnight pre-incubation, the medium was replaced with fresh medium containing test compounds after which the cells were incubated for another 24 h. Next, the cells were collected by trypsinization and proteins were extracted using Laemmli buffer and immediately heated at 96 °C for 15 min. Protein concentrations were assessed using a Pierce BCA Protein Assay Kit (Thermo Scientific, USA). Axin2 and survivin protein levels were analyzed by Western blotting. To this end, protein extracts (50 μg) were separated by 7.5% SDS-PAGE (Bio-Rad, USA) after which the proteins were transferred to nitrocellulose membranes. Subsequently, the membranes were blocked with 10% skimmed milk and incubated with a primary rabbit polyclonal antibody directed against Axin2 or a primary mouse monoclonal antibody directed against survivin (Santa Cruz Biotechnology, USA). β-actin served as a loading control. After washing, the membranes were incubated with an alkaline phosphatase-labeled anti-rabbit or anti-mouse secondary IgG antibody (Santa Cruz Biotechnology, USA) and visualized using a BCIP/NBT AP Conjugate Substrate Kit (Bio-Rad, USA). Determination of the relative intensities of the bands was performed using Quantity One software and the values were calculated as relative absorbance units (RQ) per mg protein.

### Reporter assay

CAL27 and FaDu cells were seeded in 96-well plates (1.5 × 10^4^/well) and co-transfected with 50 ng reporter (pNL(NlucP/TCF-LEF-RE/Hygro)) and 10 ng control (pGL4.54[luc2/TK] Vector) plasmids using ViaFect transfection agent (Promega, Germany) in a ratio (μg/μl) of either 1:6 (CAL27) or 1:4 (FaDu), according to the manufacturer’s recommendations. On the following day, fresh medium containing the indicated concentrations of IWP-2 or PRI-724 was added to the wells. Control cells were treated with the vehicle (DMSO). Six replicates were used for each compound, and 40 ng Wnt3a (R&D Systems, USA) was added to half of the replicates. The cells were incubated for 24 h after which chemiluminescence was detected using a Nano-Glo® Dual-Luciferase® Reporter Assay System and a GloMax microplate reader (Promega, Germany) according to the manufacturer’s protocol. The experiments were repeated twice. Relative luminescence (RL) was calculated using the formula:

$$ \mathrm{RL}=\left[\mathrm{test}\ \mathrm{compound}\ \left(\mathrm{NanoLuc}/\mathrm{Firefly}\right)\kern0.5em /\mathrm{control}\ \left(\mathrm{NanoLuc}/\mathrm{Firefly}\right)\right]\ \mathrm{x}\ 100\% $$

### Scratch wound cell migration assay

Cells (5 × 10^5^/well) were seeded in 24-well plates and grown in serum-reduced medium overnight. On the following day confluent cell layers were scratched with a tip and wells were rinsed with warm PBS buffer in order to remove detached cells. Next, fresh serum-reduced medium containing the indicated concentrations of the test compounds was added and each well was photographed using a JuLI FL miscroscope (NanoEntek, Korea). Photographs of the same areas were taken again after 7, 13 or 18 h (t_x_). The time of incubation was different for each cell line depending on the individual cell migration dynamics so that the wound area would not become closed in vehicle-treated cells. The area covered by cells was assessed for each well at both time points using JuLI FL software and the difference in cell coverage area between t_x_ and t_0_ was calculated. The experiments were repeated twice with three independent replicates per assay. Cells treated with the vehicle (DMSO) were used as controls. The relative effect on cell migration (RM) was calculated using the following formula:

$$ \mathrm{RM}=\left[\mathrm{test}\ \mathrm{compound}\ \left(\mathrm{area}\ {\mathrm{t}}_{\mathrm{x}}\hbox{--} \mathrm{area}\ {\mathrm{t}}_0\right)\kern0.5em /\mathrm{control}\ \left(\mathrm{area}\ {\mathrm{t}}_{\mathrm{x}}\hbox{--} \mathrm{area}\ {\mathrm{t}}_0\right)\right]\ \mathrm{x}\ 100\% $$

### Cell cycle analysis

Cells (1 × 10^5^/well) were seeded in 12-well plates and, after overnight pre-incubation, medium was replaced with fresh medium containing the test compounds after which the cells were grown for another 48 h. Vehicle treated cells were used as negative controls and cells treated with topotecan were used as positive controls. Next, cells were collected by trypsinization, washed with PBS buffer and fixed in 70% ethanol. After at least overnight storage at −20 °C, fixed cells were collected by centrifugation and cell cycle distributions were analyzed using a Muse Cell Cycle Kit (Merck, Germany) according to the manufacturer’s protocol. Briefly, cell pellets were washed with PBS buffer and next incubated with propidium iodide in the presence of RNase A. Flow cytometric analysis of the stained samples was performed using a Muse Cell Analyzer (Merck, Germany) and data from two independent experiments were analyzed using Muse 1.5 Analysis software.

### Apoptosis assay

Cells (1 × 10^5^/well) were seeded in 12-well plates and, after an overnight preincubation, the medium was replaced with fresh medium containing the test compounds after which the cells were grown for another 48 h. Vehicle treated cells were used as negative controls and cells treated with topotecan were used as positive controls. Next, cells were collected by trypsinization and caspase activities were measured using a Muse Caspase-3/7 Kit (Merck, Germany) according to the manufacturer’s protocol. A fluorescently labeled peptide was used as caspase substrate. The released dye interacts with DNA. Co-staining with 7-AAD allows for discrimination of cells into four groups: live, early apoptotic, late apoptotic and dead. Briefly, cell pellets were washed with PBS buffer, incubated in the presence of the substrate peptide and 7-AAD and analyzed by flow cytometry using a Muse Cell Analyzer. Two independent experiments were performed and data were analyzed using Muse 1.5 Analysis software.

### Statistical analysis

Student’s t test was used for analysis of the significance of differences between experimental groups and their respective controls, with *p* ≤ 0.05 considered as significant. The analyses were performed using STATISTICA 10 software.

## Results

### Effect of gene knockdown on the expression of Wnt target genes

**Fig. 1:**
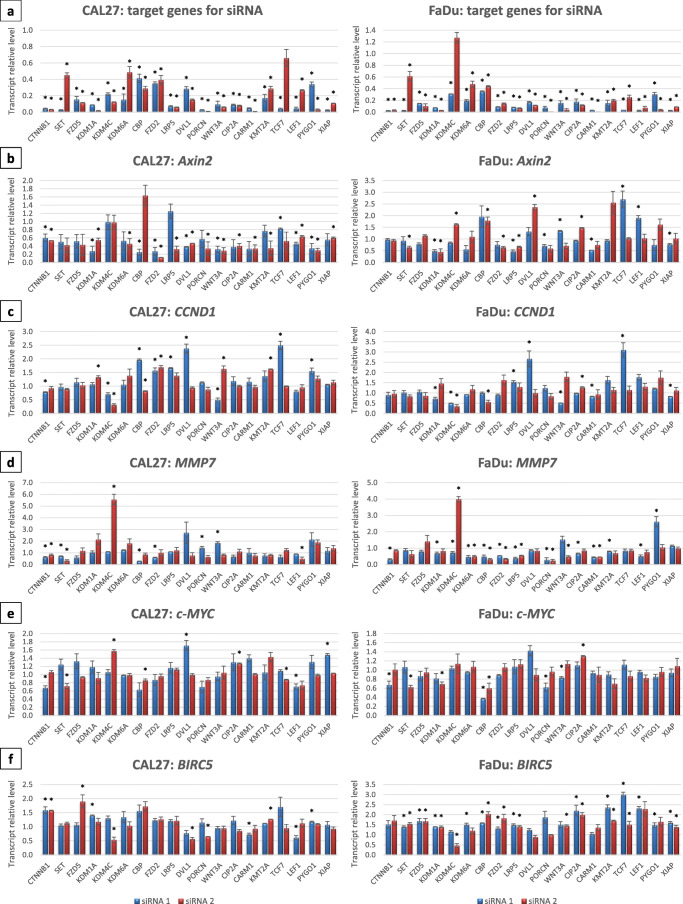
Effects of targeted knockdown of selected genes on β-catenin-dependent gene expression in CAL27 and FaDu cells. Two distinct siRNA molecules were used for the silencing of each gene and the results are presented independently for each siRNA. Mean values ± SD from two experiments are shown. **a** Efficiency of target gene knockdown mediated by a 48-h transfection of cells with each siRNA evaluated by qRT-PCR. **b-f** Effect of each siRNA on the transcript level of Wnt pathway target genes: *Axin2*, *CCND1*, *MMP7*, *c-MYC*, *BIRC5*. Asterisks above bars denote statistically significant changes, *p* ≤ 0.05

The canonical Wnt cascade represents a multistep pathway that can be inhibited at many different levels, starting with ligand secretion and ending with TCF/LEF-dependent transcription activation. We have selected multiple druggable targets (PORCN, WNT3A, FZD2, FZD5, LRP5, DVL1, CIP2A, SET, KDM1A, KDM4C, KDM6A, CBP, CARM1, KMT2A, TCF7, LEF1, PYGO1, XIAP) and performed a siRNA screen to identify those targets of which knockdown significantly inhibits activity of the Wnt pathway. To this end, we assessed the expression level of TCF/LEF target genes: *Axin2*, *CCND1*, *MMP7*, *c-MYC* and *BIRC5*. Two distinct siRNA duplexes were used separately for the knockdown of each gene. First, we evaluated whether the transfection conditions allowed for efficient gene silencing. In most of the cases, we found that siRNA transfection of CAL27 and FaDu cells led to a significant decrease (≥ 70% reduction) in the level of the gene transcript targeted by the siRNA duplex (Fig.1a). In order to next evaluate whether our experimental system allows for the identification of target proteins for Wnt inhibition, we assessed the activity of siRNA duplexes against *CTNNB1*, which encodes β-catenin, the key protein in the activation of Wnt pathway-dependent gene expression. We found that targeting *CTNNB1* reduced the level of *Axin2*, *MMP7* and *c-MYC*, indicating that the experimental set-up is suitable for performing the screen. Next, the expression level of Wnt pathway-dependent genes was analyzed 48 h post-transfection. We found that the expression of *Axin2* and *MMP7* was more frequently affected than that of the other Wnt target genes (Fig. 1, b-f). siRNAs targeting histone demethylases (KDM1A, KDM4C, KDM6A), methyltransferases (KMT2A, CARM1) and acetyltransferases (CBP) elicited a significant inhibition of TCF/LEF-dependent gene expression. Such an effect was also observed after transfection with siRNAs against Porcupine, Dishevelled, Wnt3a and Frizzled 2. Based on the results obtained, we selected eight targets for further analysis of their potential to inhibit canonical Wnt signaling: Porcupine, Dvl, KDM1A, KDM4C, KDM6A, KMT2A, CARM1 and CBP. Small molecule inhibitors are commercially available for all of these proteins.

### Cell viability analysis

**Fig. 2:**
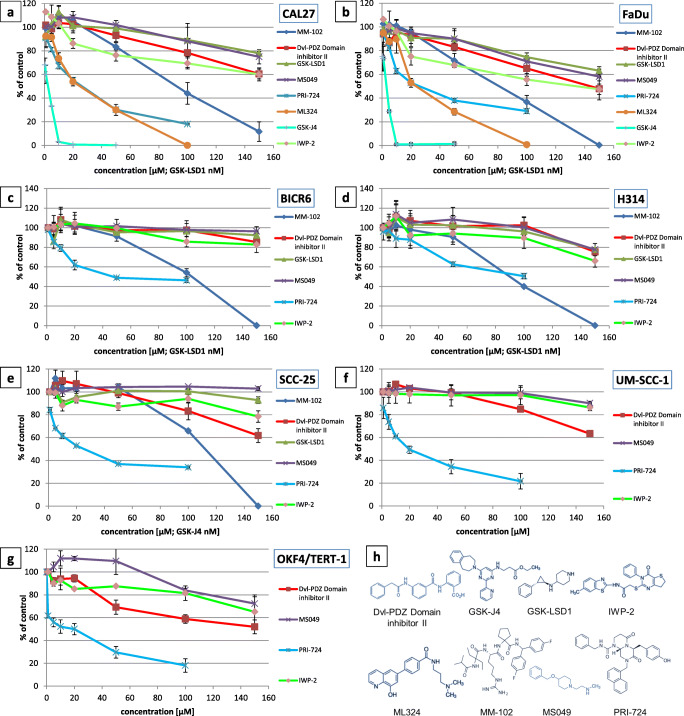
Effect of small molecule inhibitors of target proteins on the viability of head and neck carcinoma and immortalized oral keratinocyte cell lines after a 24-h treatment (**a**-**g**). The results are mean values from two independent experiments ± SD. The chemical structure of the studied compounds is shown in panel (**h**)

In order to choose appropriate concentrations of the inhibitors for subsequent analysis, their effect on the viability of HNSCC and immortalized keratinocyte cell lines was assessed using a resazurin assay (Fig. 2). We found that inhibitors of KDM6 (GSK-J4), KDM4 (ML324) and CBP/β-catenin (PRI-724) led to the strongest reduction in cell viability. The inhibitor of KMT2A (MM-102) exerted a significant decrease in cell viability at high concentrations. The inhibitors of Dishevelled (Dvl-PDZ Domain Inhibitor II), Porcupine (IWP-2), CARM1 (MS049) or KDM1A (GSK-LSD1) showed low to moderate effects on cell viability within the range of the tested concentrations. Sub-toxic concentrations (leading to the reduction in cell viability by ≤50%) were chosen for each compound and used in subsequent experiments.

### Effect of the tested compounds on the expression of β-catenin target genes

**Fig. 3:**
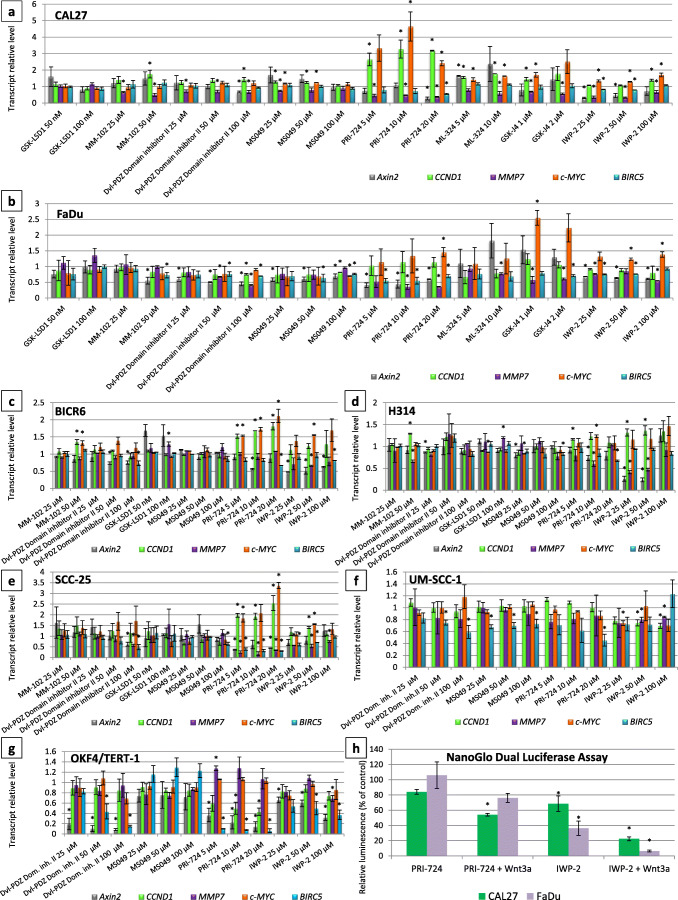
Effect of small molecule inhibitors of target proteins on the transcript level of Wnt pathway target genes: *Axin2*, *CCND1*, *MMP7*, *c-MYC*, *BIRC5* in head and neck carcinoma (**a-f**) and immortalized oral keratinocyte (**g**) cell lines after a 24-h treatment. Panel (**h**) presents the results of the reporter assay measuring the effects of IWP-2 and PRI-724 on TCF/LEF-dependent luciferase expression in CAL27 and FaDu cells. Mean values ± SD from two independent experiments are shown. Asterisks above bars denote statistically significant changes, *p* ≤ 0.05

**Fig. 4:**
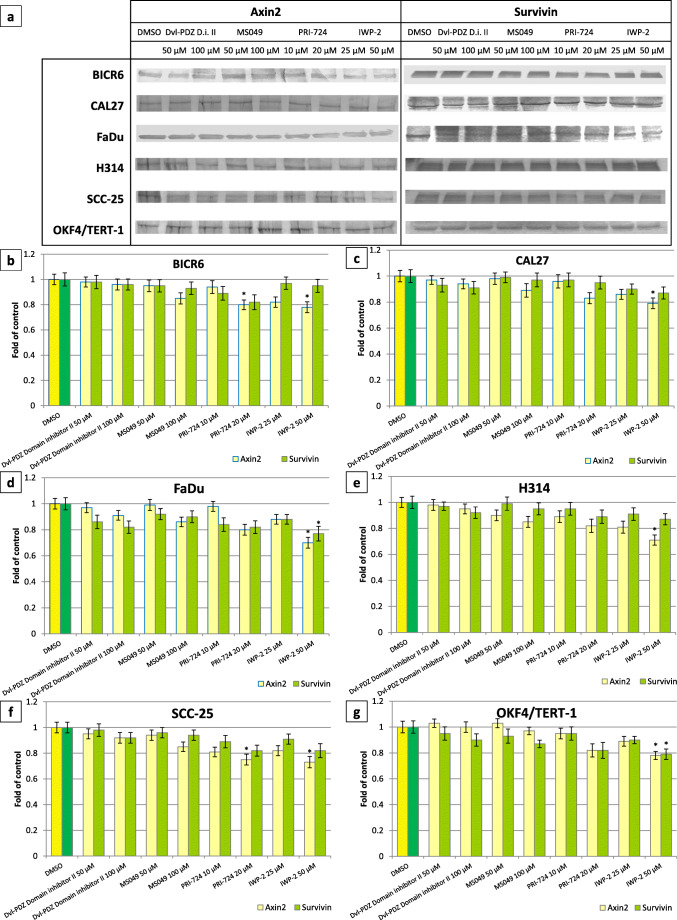
Effect of selected chemicals on Axin2 and survivin protein levels after a 24-h treatment. Representative electrophoregrams (**a**) and the results of the assessment of the level of Axin2 and survivin in total cell extracts by Western blotting (**b-g**) are presented. Mean values ± SD from two independent experiments are shown. β-actin was used as a loading control (not shown). Asterisks above bars denote statistically significant changes, *p* ≤ 0.05

We further narrowed down the set of drug targets showing potential to inhibit Wnt-dependent transcription in three steps. First, the effect of the compounds on the level of expression of Wnt target genes was assessed in CAL27 and FaDu cells, which were also used for the initial siRNA screen (Fig. 3a, b). We found that the inhibitors of KDM4 (ML324) and KDM6 (GSK-J4) did not show the desired effects (i.e., both compounds either did not change or even induced the level of expression of *Axin2*, which is the most sensitive biomarker of Wnt pathway activity) and these were thus excluded from further analyses. The inhibitor of KDM1A (GSK-LSD1) did not show any significant activity, but it tended to slightly reduce *Axin2* expression in FaDu cells. All other compounds showed at least some potential to decrease the transcript level of Wnt target genes in one or both cell lines. Of all compounds, the inhibitor of the interaction between CBP and β-catenin (PRI-724) showed the strongest activity, i.e., it significantly reduced the level of expression of *Axin2*, *MMP7* and *BIRC5*, although it also increased the expression of *CCND1* and *c-MYC*. Similar but weaker effects were obtained by IWP-2. The inhibitors of KMT2A (MM-102), Dishevelled (Dvl-PDZ Domain Inhibitor II), CARM1 (MS049) were found to be active in FaDu, but not in CAL27, cells. These six compounds were further tested in three additional HNSCC cell lines (BICR6, H314, SCC-25) in order to assess the generality of their Wnt pathway-inhibitory effects (Fig. 3c–e). Based on the lack of desired activity in these cell lines, the inhibitors of KMT2A (MM-102) and KDM1A (GSK-LSD1) were discarded. The effects of the remaining four compounds were verified in another carcinoma cell line (UM-SCC-1) and in normal immortalized keratinocytes (OKF4/TERT-1) (Fig. 3f, g). Overall, we found that the inhibitors of CBP/β-catenin (PRI-724) and Porcupine (IWP-2) showed the strongest reductions in the levels of three Wnt target genes: *Axin2*, *MMP7* and *BIRC5*. The UM-SCC-1 cell line was found to be most resistant to the inhibitory effects of the studied compounds. On the other hand, we found that inhibition of TCF/LEF-dependent gene expression was not restricted to carcinoma cells, as they were also detected in OKF4/TERT-1 keratinocytes. The studied compounds also reduced the Axin2 and survivin protein levels (Fig. 4). The effect of the two strongest modulators of Wnt pathway-dependent transcription were further verified in CAL27 and FaDu cells using a TCF/LEF luciferase reporter assay (Fig. 3h). To this end, the cells were treated with PRI-724 or IWP-2 for 24 h in the presence or absence of Wnt3a ligand. We found that IWP-2 was more effective than PRI-724 and led to a significant reduction in the TCF/LEF-dependent level of luciferase production. The effects were even more pronounced when the cells were further stimulated with Wnt3a.

### Effect of selected inhibitors on cell migration, cell cycle progression and apoptosis

**Fig. 5:**
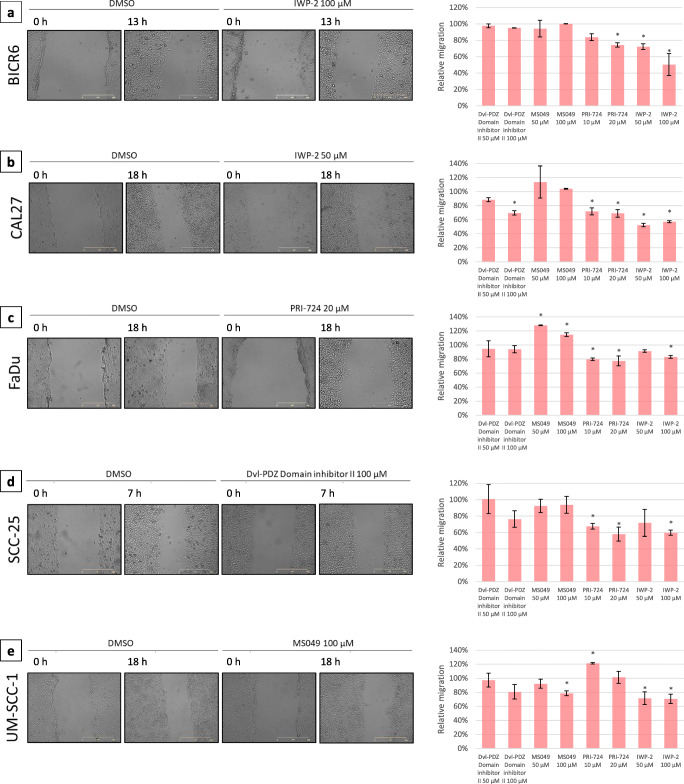
Effect of selected chemicals on cell migration assessed by scratch wound healing assay. Confluent cell layers were wounded with a pipette tip and further grown in serum-reduced medium for the indicated times, which were different for each cell line because of different proliferation dynamics. Representative microscopic photographs of the scratched areas taken initially and at the endpoint are shown on the left. Relative cell migration was calculated by comparing the change in the area covered by cells resulting from the treatment with the tested compounds with the results obtained with vehicle-treated cells. The respective graphs on the right present mean values ± SD from two independent experiments. Asterisks above bars denote statistically significant changes, *p* ≤ 0.05

**Fig. 6:**
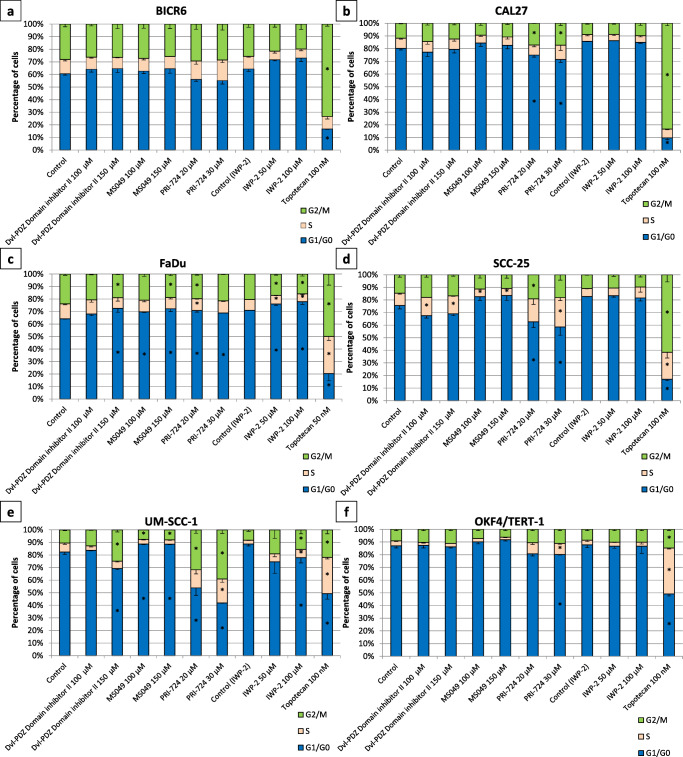
Effect of selected compounds on cell cycle distribution after a 48-h treatment. The graphs present the percentage of cells in G1/G0, S or G2/M phase measured by flow cytometry after propidium iodide staining. Mean values ± SD from two independent experiments are shown. Topotecan was used as a positive control. Because of the limited solubility of IWP-2 and the necessity of using higher concentrations of DMSO (vehicle), a separate control was included for this compound. Asterisks denote statistically significant changes, *p* ≤ 0.05

**Fig. 7:**
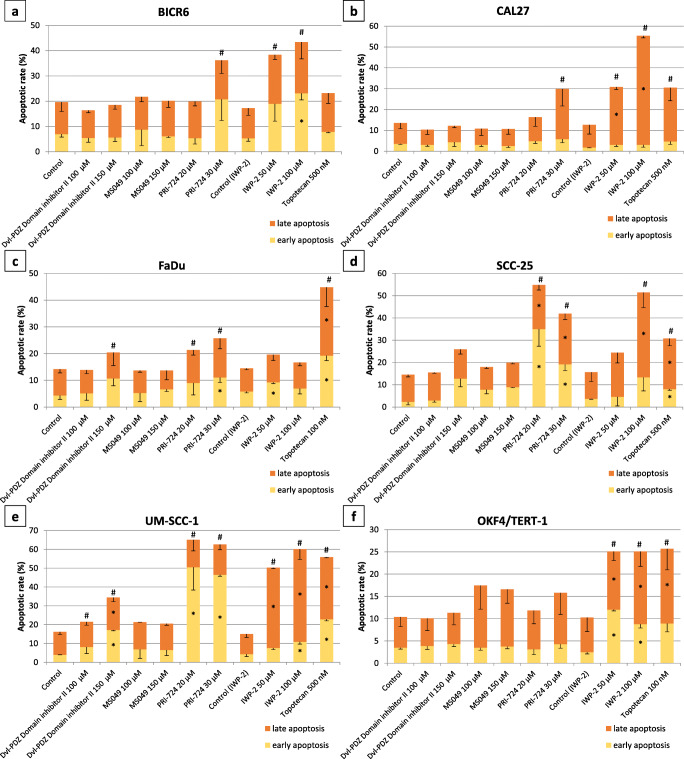
Results of the analysis of the effect of selected compounds on apoptosis after a 48-h treatment. The graphs present the percentage of cells in early or late apoptosis assessed by flow cytometry measurement of caspase 3/7 activity. Mean values ± SD from two independent experiments are shown. Topotecan was used as a reference pro-apoptotic chemical. Because of the limited solubility of IWP-2 and the necessity of using higher concentrations of DMSO (vehicle), a separate control was included for this compound. Asterisks denote statistically significant changes in the percentage of either early or late apoptotic cells, whereas hashes denote statistically significant changes in the percentage of total apoptotic cells, *p* ≤ 0.05

In order to better characterize the biological activity of the four selected inhibitors (PRI-724, IWP-2, MS049 and Dvl-PDZ Domain Inhibitor II) we performed cell migration, cell cycle and apoptosis assays. Cell migration was analyzed using a scratch wound healing assay and, by doing so, we found that PRI-724 and IWP-2 showed ability to inhibit the migration of BICR6, CAL27, FaDu and SCC-25 cells, while only IWP-2 inhibited the migration of UM-SCC-1 cells (Fig. 5). The compounds also showed a low to moderate ability to change cell cycle phase distribution (Fig. 6). The strongest effects were observed for PRI-724, which reduced the percentage of G0/G1 cells in the CAL27, SCC-25 and UM-SCC-1 cell lines, but slightly increased the percentage of G0/G1 cells in the FaDu cell line. Importantly, none of the compounds significantly affected cell cycle progression in control OKF4/TERT-1 keratinocytes apart from the highest concentration of PRI-724. Topotecan was used as positive control and, as expected, led to G2/M arrest in all cell lines tested. Finally, the effect of the 4 compounds on the occurrence of apoptosis was assessed by flow cytometric measurement of caspase-3/7 activity (Fig. 7). Topotecan, which was used as positive control, led to a significant increase in the number of apoptotic cells in all cell lines tested, except BICR6. Again, PRI-724 and IWP-2 showed strongest activity. BICR6 and CAL27 cells were found to be more susceptible to the pro-apoptotic effect of IWP-2, while FaDu, SCC-25 and UM-SCC-1 cells showed stronger apoptotic rates upon treatment with PRI-724. Moreover, we found that PRI-724 and IWP-2 elicited a similar or even higher level of induction of apoptosis in comparison to topotecan in CAL27, SCC-25 and UM-SCC-1 cells. On the other hand, we found that only IWP-2 significantly induced apoptosis in control OKF4/TERT-1 keratinocytes and that the effect was similar to that elicited by topotecan.

## Discussion

Aberrations in canonical Wnt signaling are a known hallmark of colorectal carcinogenesis due to mutations in the *APC* and *CTNNB1* genes [27]. Activation of this pathway has also been observed in other types of cancer, including head and neck cancer, for which it has recently been suggested as a promising therapeutic target [28, 29]. Activation of canonical Wnt signaling is mediated by β-catenin, which activates the transcription of target genes, such as *CCND1, c-MYC, MMP7* or *survivin*, which enhance cell cycle progression and cell migration, and inhibit apoptosis [30]. Up-regulation of gene transcription may require the activity of epigenetic modifiers, such as histone methyltransferases, histone acetyltransferases or histone demethylases, and it has been reported that β-catenin-driven activation of the expression of genes associated with cell proliferation requires the presence of CBP acetyltransferase [31].

Here, we aimed to identify the most suitable targets for the inhibition of canonical Wnt signaling in HNSCC cells. A broad set of potential targets was initially investigated using siRNA-mediated gene expression knockdown. This set encompassed genes associated with initiation of this signaling pathway (*PORCN, WNT3A, FZD2, FZD5, LRP5, DVL1, CIP2A, SET*) and genes associated with nuclear regulation of β-catenin transcriptional activity (*KDM1A, KDM4C, KDM6A, CBP, CARM1, KMT2A, TCF7, LEF1, PYGO1, XIAP*). Various mechanisms may contribute to activation of Wnt signaling in HNSCC, thus, potentially both upstream and downstream proteins may serve as suitable targets for down-regulation of this cascade. In order to assess pathway inhibition, expression levels of *Axin2*, *MMP7*, *BIRC5* as well as *c-MYC* and *CCND1*, were used as pharmacodynamic readouts, similar to other recent studies [24, 32–34]. Interestingly, cell membrane-bound Porcupine and nuclear CBP emerged as the best targets for the effective down-regulation of Wnt-dependent gene expression in the HNSCC cells tested. Porcupine is an *O*-acyltransferase specific for Wnt ligands which allows their secretion, while CBP is a crucial component of the β-catenin transcriptional complex. Recently, it has been found that LGK974, which is an inhibitor of the enzymatic activity of Porcupine, significantly reduced β-catenin-dependent *Axin2* expression in approximately one third of 96 head and neck cancer cell lines analyzed [24]. Moreover, this compound was found to reduce Wnt/β-catenin pathway activation in papillomavirus-driven cutaneous squamous cell carcinomas [35]. IWP-2, another Porcupine inhibitor which has a chemical structure different from LGK974, was tested in our study. Previously, this compound has been found to effectively reduce Wnt/β-catenin signaling in gastric cancer cells [32]. In our study, IWP-2 not only significantly reduced the transcript level of *Axin2*, *MMP7* and *BIRC5* in most cell lines analyzed, but its inhibitory activity was also confirmed using a reporter assay in FaDu and CAL27 cells. Interestingly, FaDu cells have, in contrast to CAL27 cells, been classified as unresponsive to LGK974, but this conclusion was based on a stringent condition of at least 50% reduction in *Axin2* mRNA level [24]. In our study, FaDu cells showed moderate reductions in the expression levels of *Axin2*, *MMP7* and *BIRC5* after treatment with IWP-2. A stronger inhibitory effect was observed in the reporter assay, suggesting that this cell line is responsive to Wnt signaling down-regulation via Porcupine inhibition.

PRI-724 is a second generation antagonist of CBP/β-catenin interaction, which has already entered clinical trials [33]. It is a more potent enantiomer of ICG-001, which has been shown to reduce the growth of head and neck cancers in preclinical experiments [34, 36, 37]. In our study, PRI-724 reduced the level of expression of β-catenin-dependent genes, but it had a weaker effect on the production of TCF/LEF reporter-based luciferase than IWP-2. ICG-001 has been shown to specifically affect a subpopulation of stem-like tumor propagating head and neck carcinoma cells, but it has been suggested that this compound is rather cytostatic [34] and does not induce cell death. Our results point at both cytostatic and cytotoxic effects of PRI-724 in HNSCC cells. This notion was reflected by a significant decrease in cell viability in all cell lines tested and by the induction of apoptosis in all carcinoma cell lines tested, but not in immortalized oral keratinocytes.

Contradictory to our initial hypothesis, the inhibition of other target proteins did not affect canonical Wnt signaling in HNSCC cells to a significant extent. Only the blocking of Dishevelled and CARM1 led to some effects. Although the PDZ domain of Dishevelled forms only weak interactions with Frizzled receptors, it has been suggested that its inhibition may affect Wnt signaling. On the other hand, it has been suggested that the effectiveness of this inhibition could be alleviated by an increase in the level of Wnt ligands [38]. CARM1 has been shown to participate in the formation of the β-catenin transcriptional complex [39] but it has also been shown to antagonize CBP through its direct methylation [40]. Overall, we found that the effect of Dvl-PDZ Domain Inhibitor II and MS049 on β-catenin-dependent gene expression was weak and that blocking of the PDZ domain slightly affected cell cycle distribution and induced apoptosis in HNSCC cells. These observations indicate that these strategies may not have a significant therapeutic value in this type of cancer. The inhibitory activity of the PDZ domain may be too low to effectively reduce Wnt signaling. The weak effects of MS049 may indicate that CARM1 does play a too limited role in the canonical Wnt pathway in HNSCC in order to allow therapeutic efficacy.

Recently, it has been revealed that epigenetic modifiers, including CBP, MLL and KDM6, may contribute to HNSCC initiation and progression as a result of mutation [41]. These proteins may also take part in β-catenin-dependent transcriptional control. CBP has been found to co-operate with MLL (KMT2) in triggering H3K4 trimethylation at promoters of β-catenin-dependent self-renewal genes in salivary gland tumors [36]. However MM-102, which inhibits MLL activity, did not alter the level of expression of Wnt target genes in our study. KDM4 demethylases have also been found to be required for β-catenin transcriptional activity in gastric and colorectal cancer cells [42, 43], but their inhibition with ML324 did not show any effects on HNSCC cells in our study. LSD1/KDM1A histone demethylase has also been implicated in head and neck carcinogenesis [44], and it has recently been reported that a GSK-LSD1 inhibitor may down-regulate β-catenin signaling only in myoepithelial and not in tonsil cancer cells [45]. These observations are in agreement with our results.

Collectively, our data show that targeting Porcupine or CBP/β-catenin interaction may serve as a suitable strategy for the inhibition of canonical Wnt signaling in HNSCC cells. We found that the activity of IWP-2 and PRI-724 is associated, not only with the inhibition of cell proliferation, but also with the induction of apoptosis. In addition, we found that both compounds affected cell migration to a certain extent, which is in line with observations that Wnt signaling is related to cancer cell invasion. As yet we cannot, however, exclude that the effects observed upon HNSCC cell treatment with IWP-2 and PRI-724 may also be related to the modulation of other pathways besides the canonical Wnt pathway. Further studies are required to elucidate in detail the modes of action of these compounds and to provide direct evidence for their anti-cancer action in other pre-clinical models.
